# Progress in State Medicine

**Published:** 1897-04-10

**Authors:** 


### Progress in State Medicine.

**Purification of Sewage.**—The object of the various systems of sewage treatment at present employed is by the addition of chemical agents to precipitate the organic matters in suspension and solution, and to obtain an effluent as nearly sterile as possible. Grether[1] demonstrates that the addition of large quantities of lime, exceeding the amounts that can practically be employed, fails to disinfect sewage, and that when highly diluted with river waters there is no proportionate reduction in the number of organisms. Increasing importance is now attached to the treatment of sewage by biological methods. No attempt at sterilisation is made, but all processes are subordinated to the requirements of organisms, which by their growth effect the desired change in the sewage. Two such processes have recently been given an extended trial.

The septic tank system[2] has been experimentally tested at Yeovil and Exeter. The sewage, after rough straining, passes into a large covered tank, where, deprived of light and air, it is acted upon by anaërobic organisms. There is little sedimentation or formation of gas, and the process of conversion is completed by passing the effluent through aërating troughs and coke filters. The Exeter sanitary authorities have applied to the Local Government Board for permission to adopt this process, and an inquiry has been ordered. On two previous occasions, at Rushden and Staines, the Local Government officials have withheld their sanction.

Thudicum[3] describes the experimental work undertaken by the London County Council at Sutton. Beds of burned ballast, 4 ft. in thickness, are here employed. To these beds is added a culture of the micrococcus candicans, an organism isolated by Dibden from coke-breeze filters.

Sewage pumped on to the beds is allowed to remain for two hours; the organic matter present is oxidised by the micro-organisms, and after filtration through land the effluent is in a fit condition to be turned into watercourses. The life of the purifying beds is practically unlimited. Each is used twice daily, and it is calculated that one acre of filter can purify 10,00,000 gallons of sewage per diem, requiring only a brief time for recovery. The capacity of the filter may be increased either by increasing the surface, or the depth, for the action is not confined to the surface layers. Both these methods aim at destroying organic matter in Nature's own way, namely, by the work of bacteria. Should they prove satisfactory a great economy would be effected, for the use of chemicals, settling tanks, and much pumping machinery can be dispensed with.

**Water Filtration.**—In connection with the London water supply, much attention has lately been drawn to the uncertainties and risks attendant upon the use of gravel filter-beds as at present constructed. In India, owing to the presence of cholera, the dangers of a polluted water supply are still more serious, and the installation at Darjeeling of a Pasteur-Chamberland filter of sufficient size to deal with the town's supply will doubtless be followed by a similar departure in many other localities.[4] Each filter consists of an iron cylinder 3 ft. high and 18 in. long, and contains 150 candles. The inlet is above, and the cover is moveable to permit of cleaning. The pressure in the cylinders is equal to about two atmospheres. The interior is cleaned by washing with a 10 per cent. solution of hydrochloric acid. The supply is 150,000 gallons per diem. To prevent the possibility of failure owing to cracks in the porcelain or imperfections in the fittings, each cylinder is periodically tested. In the process of testing, advantage is taken of the fact that air cannot pass through candles made of diatomaceous earths, although water passes easily. A pressure-gauge is fitted to the cylinder, the cover fixed, and air forced in under pressure. The height of the gauge is then noted, and any subsequent fall indicates the presence of some crack or fissure through which air is escaping, and through which it would, of course, be possible for unfiltered water to pass.

In public offices and in private houses it is still extremely common to see filters other than those made of the porcelain clays, in spite of the fact that recent investigations have pronounced all such types to be unsatisfactory, inasmuch as they not only fail to separate micro-organisms, but, after a longer or shorter period of use, add very considerably to the number already present. Sims Woodhead[5] and Cartwright-Wood, in verification of the work of Plagge, of Berlin, found that three filters only—the Pasteur-Chamberland, Porcelaine d'Amante, and Berkfeldt—appeared to protect against the communication of infectious diseases through the agency of water. These, it was recommended, should be used as pressure-filters, for in this way a supply could be speedily obtained sufficient for all domestic purposes.

**School Inspection.**—An increasing amount of attention is being paid in the United States to the question of school inspection, both with a view to the early detection of cases of infectious disease and with the purpose of forming special classes for pupils who, owing to mental or physical defects, may require particular attention. The city of Boston[6] is divided for this purpose into 50 districts, to each of which a medical man is attached. Each morning the junior teachers report any case of sickness to the head master of the school, who in his turn reports to the visiting physician when making his daily visit. It is not part of the duty of the medical man to prescribe, but he may examine the pupil and must make proper arrangements for his removal and isolation, if required. A similar system is now about to be adopted in New York. In England special classes for the training of defective children are established in London, Bristol, and many other large towns. No attempt, however, has been made to detect the early cases of infectious disease, or to provide skilled assistance for the discovery of defective sight and hearing. Of 53,000 children examined recently in the public schools of Baltimore[7] 57 per cent. had defective sight, and of 20 children in Liverpool denominated by teachers as "bad," Permewan found only six had normal hearing. In London Brudenell Carter found more than 50 per cent. of 8,000 children examined had defective eyesight.

The harm resulting, not only to the individual, but also to society, from the educational neglect of these children renders it most desirable that the State should institute some scheme of school inspection, which should be carried out by specially qualified medical officers.

[1] Archiv. f. Hygiene XXVII., 3. [2] Lancet, Dec. 5, 1896. [3] Sanitary Record, Dec. 18, 1896, "The Ultimate Purification of Sewage." [4] American Journal of Medical Sciences, Feb. 1897. [5] B. M. J., 1894, "On Domestic Filters." [6] Journal American Med. Assoc. [7] Brooklyn Medical Journal, Feb., 1897, "Report of County of King's Committee."

